# ‘If you can’t make it, you’re not tough enough to do medicine’: a qualitative study of Sydney-based medical students’ experiences of bullying and harassment in clinical settings

**DOI:** 10.1186/s12909-020-02001-y

**Published:** 2020-03-24

**Authors:** Laura Colenbrander, Louise Causer, Bridget Haire

**Affiliations:** The Kirby Institute, UNSW Sydney, Wallace Wurth Building, High Street, Kensington, NSW 2052 Australia

**Keywords:** Medical students, Bullying, Harassment, Culture, Hierarchy

## Abstract

**Background:**

Media exposés and academic literature reveal high rates of bullying and harassment of medical students, most commonly by consultant physicians and/or surgeons. Recent reports reveal the medical profession to be characterised by hierarchy, with verbal abuse a ‘rite of passage’, as well as sexist and racist behaviours.

**Methods:**

Semi-structured in-depth interviews were conducted with ten current or recently graduated medical students from Sydney-based medical schools. Interviews were audio-recorded, transcribed verbatim, and thematically analysed.

**Results:**

Hierarchy, and a culture of self-sacrifice, resilience and deference, were identified as problematic elements of the medical profession. In the minds of participants, these factors created barriers to reporting mistreatment, as participants felt reporting led to being labelled a ‘troublemaker’, affecting career progression. Additionally, participants stated that avenues of recourse were unclear and did not guarantee confidentiality or desired outcomes.

**Conclusions:**

Mistreatment is continuing in clinical teaching and has negative consequences on medical students’ mental health and learning. Structural change is needed to combat institutionalised mistreatment to ensure the wellbeing of future doctors and high quality patient care.

## Background

Hierarchy plays an essential role in all professions. However, hierarchy in medicine today is characterised by an imbalanced relationship between superior and subordinate, rather than a mentoring relationship between teacher and learner [1]. While the culture of patient care is changing to incorporate a more patient-centred perspective [2], the demands of busy, unpredictable clinical workplaces mean that strict power hierarchies continue to govern hospitals, and professional culture is dominated by power imbalance, overworking and subsequent ‘burnout’.

The lingering presumption that mistreatment in this environment is ‘beneficial’ because ‘if you can’t make it, you’re not tough enough to do medicine’ [3] has been challenged by exposés of workplace bullying and harassment. Cases of doctors being bullied out of specialty training programs, hospitals stripped of training accreditation, and declining mental health of medical trainees, are all purportedly products of the exigent culture within medicine [4–8]. In the past year alone, Bankstown-Lidcombe, St George, Royal Prince Alfred, Westmead and Tamworth hospitals have all made headlines regarding mistreatment of junior doctors [8–11]. The umbrella term ‘mistreatment’ will be used to refer to professional misconduct that occurs in clinical settings, and definitions of bullying, harassment and sexual harassment can be found in Appendix A [12].

### Rates of bullying & harassment in medicine

In 1990, The Journal of the American Medical Association (JAMA) published a landmark study observing the incidence, severity and significance of abuse of medical students [13]. This United States (US)-based study was the first to document the incidence, severity and significance of abuse from medical students’ perspectives, and thus provides important background for our study. The anonymous cross-sectional questionnaire revealed 46.4% of the 519 participants reported being mistreated at some point during medical school, 80.6% of seniors were abused by their senior year, while 49.6% of those abused stated that the most serious episode of abuse would ‘always affect them’.

Medical schools and colleges have since instituted anti-harassment policies, grievance committees, ‘confidential’ complaint avenues and counselling programs [6, 10, 14]. Despite such efforts to ameliorate abuse and increase awareness, media exposés of mistreatment in medical training remain frequent.

Academic literature confirms ongoing mistreatment of medical students in medical schools overseas. A 2006 US study, which included 2316 participants from sixteen US medical schools, reported 85% of students being ‘harassed or belittled’, 40% experiencing both, and 13% describing an incident as severe [15]. A 2014 systematic review and meta-analysis of fifty-seven cross-sectional studies and two cohort studies reported 59.4% of medical trainees, globally, had experienced at least one form of harassment or discrimination [16]. Both studies identified verbal harassment as the most common form of mistreatment, and consultants as the most consistent perpetrators. While these data cannot be extrapolated to the Australian context, they are suggestive of an issue within the culture of medicine that might transcend national boundaries.

In Australia, the Australian Medical Association (AMA) confirmed that more than 50% of doctors and trainees (not including medical students) have been bullied or harassed, with verbal harassment and consultants again most commonly cited [17]. Overall, the literature reveals consistently high (at least 50%) rates of bullying, harassment and discrimination in medical training [12, 18–22]. This incidence is alarmingly high, despite authors noting probable underreporting due to studies being led by faculty members of students’ institutions.

### Students’ perceptions of mistreatment

A 2015 pilot study involving surveys of final stage medical students from two Australian medical schools found that medical students believe mistreatment is institutionalised [22]. Participants perceived doctors as having leeway to bully because the medical profession is characterised by a ‘different moral order’ [22]. This protection allows abusive behavior, unacceptable in any other professional environment, towards patients, staff, and students.

One of the difficulties in quantifying the extent of mistreatment of medical students is the diversity of students’ perceptions of mistreatment. Surveys asking for specific instances of abuse, such as sexual harassment or racism, narrow participants’ perceptions of their own mistreatment and impose preconceived definitions of abuse, which may lead to underreporting. A US-based study found 73% (*n* = 4530) of nurses reported witnessing disruptive behaviour within their hospital, compared to 48% of physicians [23]. The lack of registering mistreatment as problematic within medical professionals likely contributes towards bystander silence, and is therefore a factor that demands further research. This study aims to provide an Australian perspective on this topic.

While there exists substantial literature on the *rates* of bullying and harassment within medical schools, much of the available data lacks students’ *perceptions* of mistreatment. This qualitative study captures personal stories, the standard of medical teaching, and how experiences have impacted medical students, which quantitative data fails to represent. Additionally, it systematically analyses this information in a way that media exposés cannot, allowing previously unappreciated patterns to emerge.

### Aims

This study aimed to capture medical students’ experiences of bullying, harassment, culture and teaching in clinical settings, as well as the effects of mistreatment on students. Additionally, the study aimed to explore the factors contributing to underreporting; do students fail to recognise behavior as problematic, or do they harbour distrust in the complaint system? The key objective is to provide a rich account of Sydney-based medical students’ perceptions of mistreatment in clinical spaces. The study also asked medical students what changes they believe may be effective in increasing accessibility of complaint systems. The findings of this study will help inform future anti-bullying strategies and is anticipated to have a positive effect on clinical teaching.

## Methods

### Research design

Qualitative data were generated through semi-structured in-depth interviews. The interview process allowed appreciation of ‘context-specific depth’ [24] through open-ended enquiry, and the unpacking of differing perspectives behind behaviours, beliefs and assumptions [25]. Furthermore, open-ended discussion promoted stronger completion and formulation of interviewee speech by the researcher [26, 27].

### Participants

Recruitment occurred online via social media. To be eligible, participants were required to satisfy the following criteria (1) aged 18 years or above; (2) current medical student or recent graduate (within 2 years of graduating); and (3) studying at a Sydney-based medical school.

### Data collection methods

Data were collected through semi-structured, in-depth, face-to-face or telephone interviews. Informed consent was obtained prior to commencing each interview, and pseudonyms were used to preserve anonymity. The interview guide was developed from a literature review, and the semi-structured interview guide was piloted in researchers at the Kirby Institute who are medically trained. Interview domains can be found in Appendix B. Interviews were audio recorded, transcribed verbatim, de-identified and stored securely at the Kirby Institute, UNSW Sydney.

### Investigator team

All interviews were conducted and transcribed by the first author LC, who is herself a medical student. The benefits of peer interviewers identified in the literature are improved acceptance of disenfranchised groups, improved quality of research, and increased comfort of participants in the research process, while the challenges relate to the training and support of peer researchers [28]. This team also included one experienced qualitative researcher (BH) and an experienced physician/researcher (LC) in supervisory roles minimising the training burden and maximising the benefits of the peer researcher approach, where rapport and trust were quickly and effectively established with participants.

### Data analysis

**Fig. 1:**
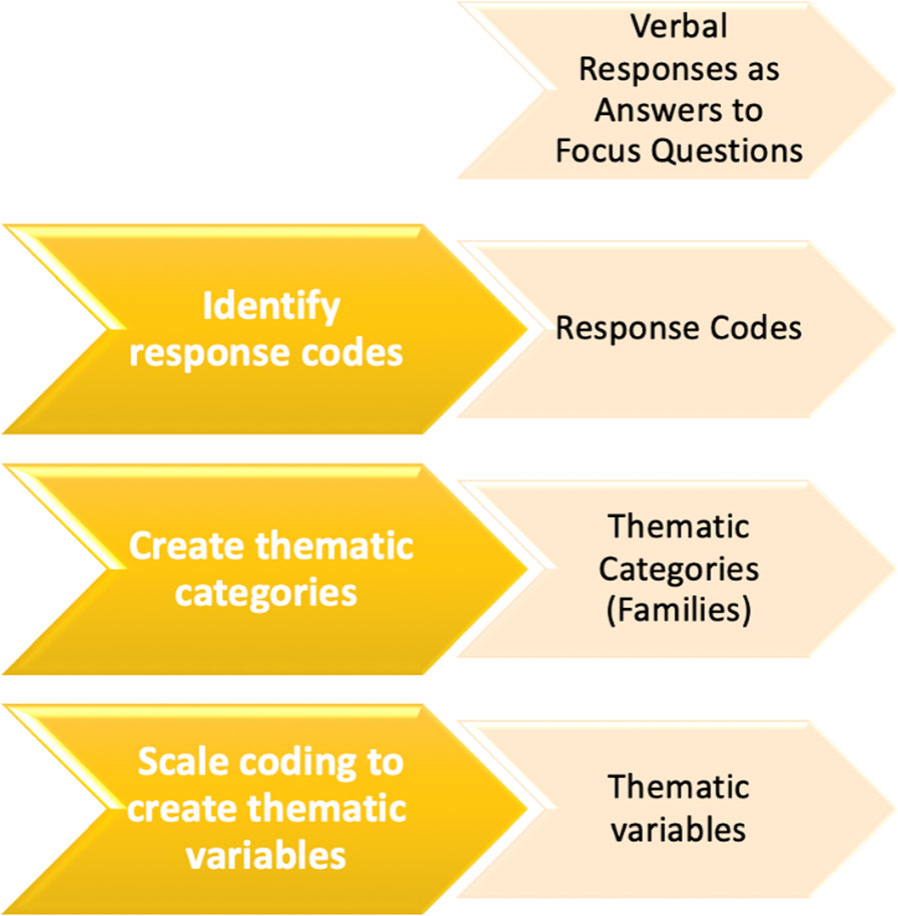
Stages of Thematic Analysis [30]

Data were managed, coded and analysed using NVivo software [29]. The identification and analysis of themes occurred in a ‘bottom-up’ manner, in that data was not coded to fit into the pre-existing research question framework. This thematic analysis was conducted using a systematic framework developed by Castro, Kellison [30] adapted from Braun and Clarke [31] (Fig. 1). There were six steps in the analysis: 1. close reading of interview transcripts; 2. generating initial codes through inductive process; 3. discussion within the research team of initial coding to identify thematic categories; 4. review and revise initial thematic categories and coding with respect to the whole data set; 5. defining themes; 6. analysis of the themes and selected quotes in relation to the whole data set.

## Results

The participant sample comprised ten current or recently graduated medical students (three men and seven women) of metropolitan, rural and international backgrounds (see Table 1).

**Table 1:** Participant Demographics

Participant Pseudonym	Age range	Gender	Ethnicity	Year of Study	Geographic Origin
Colette	26–30	Female	Caucasian	Three	Metro
Alice	18–21	Female	Caucasian	Four	Metro
Theo	18–21	Male	Caucasian	Four	Metro
Sanjna	22–25	Female	BAME^a^	Graduated 2018	Metro
Arosh	22–25	Male	BAME^a^	Six	International
Peter	18–21	Male	Caucasian	Four	Rural
Abby	22–25	Female	Caucasian	Five	Metro
Lily	22–25	Female	Caucasian	Two	Metro
Jarra	22–25	Female	Caucasian	Four	Rural
Lauren	22–25	Female	Caucasian	Two	Metro

^a^BAME refers to Black, Asian and people from minority ethnic backgrounds

Participants’ responses generated six thematic categories for analysis: Hierarchy, culture, avenues of recourse, pressures on medical students, mistreatment from patients and quality of teaching. Together, these thematic categories provide insight into how medical students in this study perceive that the structures and cultures of clinical settings collude to enable mistreatment to occur in an environment where speaking out against it is considered likely to have a negative rebound effect on the whistle-blower.

### Hierarchy

All ten participants described the ‘hierarchy’ as a prominent feature of medical culture. Participants stated the hierarchy was necessary to an extent, providing a chain of command in which junior defers to senior in complex medical decisions.

*‘You do need a hierarchy, or at least an identifiable person who is overall responsible… You’re there for the patients, and the patient’s deteriorating… You do need someone who’s like, “This is what we’re going to do”.’ (Peter)*

However, participants noted a toxic workplace culture from abuse of the hierarchy. They described ‘servant’ demands of medical students and trainees, including: demanding coffee (then complaining it’s not right); not allowing students to microwave their food; and forcing one student to carry handwash rather than taking notes, which made that student feel ‘completely disrespected’ (Arosh).

Participants said they did not feel able to report issues because of the hierarchy. Senior doctors were overwhelmingly considered unapproachable because they were ‘self-important’, sexist, uninterested, too busy, or participants feared verbal abuse. All three male participants, but only one female participant, while intimidated, stated they overcame this and asserted their right to approach seniors.

Participants felt that underreporting contributed to lack of intergenerational change. They suggested more ‘top-down’ methods of intervention were necessary to challenge the cyclical nature of mistreatment, currently a ‘rite of passage’, whereby the bullied become the bullies.

### Culture

Four key themes were generated in the discussion of medical culture. The first overarching theme identified in all transcripts was the concept that self-sacrifice was a value inherent to the medical profession. Doctors and, to a lesser extent, medical students are expected to compromise their personal lives, including having children, in order to progress in their careers. Participants articulated an expectation for medicine to consume practitioners’ identities, and that sacrifice, often manifesting as overworking and underreporting, is a ‘rite of passage’ (Alice).

*‘The culture is to put medicine before everything in your life… probably from experiencing medicine, from the competitive nature of getting into medicine, the type of people who therefore get in.’ (Alice)*

There was some discord between how problematic participants viewed this issue. Some considered it outrageous, and that medicine should be considered ‘just a job’ (Peter and Sanjna), while others (Theo and Abby) said self-sacrifice may be necessary to maximise patient care.

*‘There’s always the aspect of, “If you leave early, patients don’t get care”, and if your hospital doesn’t find a way for you to be paid overtime, you still have to do it because otherwise there are very real, tangible consequences.’ (Abby)*

The second theme highlighted was when participants were asked what qualities pervade medical culture, resilience being one of the most commonly conceptualised values instilled in medical training, six participants discussing this theme.

*‘You need to shrug off, you need to compartmentalise these things, and I think we’re taught that a lot in med.’ (Arosh)*

The third theme generated was ‘imposter syndrome’: participants identified that they valued humility and said they were unwilling to ask questions or request help for fear of appearing ‘incompetent’. This was often accredited to the type of people admitted to medical school: high achievers with competitive ambitions.

*‘If you feel like you’re asking a stupid question and wasting their time… that might reflect on you, you might look stupid, you might look like you don’t know what you’re doing.’ (Lauren)*

*‘I just feel like a lot of people in medicine have imposter syndrome and are constantly like, “I’m not good enough” and “I didn’t deserve to get in”.’ (Jarra)*

The fourth theme generated was the cultural practice of deference to senior doctors, and was raised when participants were asked what they thought about their clinical teachers and if they found them approachable. Recognition of their own limitations, and respect for consultants’ experience and knowledge, made participants feel like they had to go out of their way to ‘be nice’ (Alice) to senior clinicans.

*‘Whenever the registrar called the consultant, they’d always be like, “I’m so sorry for calling you, just a really quick question.” They’d apologise so profusely and I was like, “That is literally [the consultant’s] job”… and the fact that you had to hesitate that much before calling them is so bad.’ (Sanjna)*

This is one example of the excessive deference described by participants. Another was the practice of addressing the consultant as ‘Doctor’, as opposed to their name, which participants thought was outdated.

### Avenues of recourse

Seven participants felt uncertain about the avenues of recourse available to them. Barriers to reporting included insufficient assurance of confidentiality; lack of clarity regarding whether incidents fell under the university or hospital’s purview, meaning grievance escalation policies were unclear; and outcomes being characterised as inadequate.

The perceived lack of anonymity prevented reporting because of the commonly cited problem of dependency on senior doctors to ‘sign off’ on trainees and students in order to progress through their training, as well as identified whistle blowers struggling to find future employment.

*‘It’s a constantly picking game of keeping people who are important to your future on your side.’ (Abby)*

Regarding outcomes, of the four participants who had reported an incident or knew someone who had, none had experienced desired outcomes. This included ‘sexist behaviour from one of the surgeons’ (Peter), on which the clinical school had insufficient authority to act. A further five participants said that *if* they did experience bullying or harassment, they would not come forward because they do not believe any action would be taken, the clinical school does not have enough power to act, or the hospital/clinical school does not invite criticism. The remaining participant said he only believed there would be outcomes if the person reporting made enough of an ‘uproar’ (Theo).

*‘There’s a huge amount of fear and stigma about taking things to the university, also just general disillusionment that the university, that the hospitals, that the structures that are supposed to be there to support us, have failed pretty consistently, and people aren’t willing to put themselves in danger to have another go at it.’ (Abby)*

*Lily: I like to think they’d be spoken to about it in a confidential manner, and then given a warning, and then if it continues to happen, then they’d be removed from that position.*

*Interviewer: Do you think that’s actually what would happen?*

*Lily: No… I think they’re very rarely removed from that position, to the point that if they are it gets in the Sydney Morning Herald.*

Furthermore, the networking culture within the hierarchy of clinical teaching settings made participants fear acquiring a reputation of being sensitive, a troublemaker, or difficult to work with if they did not comply with demands of senior doctors, as this could have direct repercussions on academic marks or career progression.

*‘No matter how valid your complaint is, hospital management, I think, will tend to see you as the person who is a bit of a troublemaker, or caused a fuss… There might be retribution.’ (Sanjna)*

For these reasons, participants overwhelmingly said that they would only come forward when they had nothing to lose, such as if they had no interest in pursuing the specialty in which they experienced the abuse; if they could provide concrete evidence, a high burden of proof existing for medical students; or they were willing to stake their career.

*‘[It] couldn’t be interpreted as something where I was just a bit sensitive about it. It would have to be, “This is clearly not okay”.’ (Colette)*

To remedy these obstacles, three participants suggested implementation of a formal grievance escalation policy, such as the Vanderbilt Coworker Observation Reporting System (CORS).

### Pressures on medical students

Five main factors were identified as causing stress to medical students. The first was academic pressure, with eight participants stating how difficult it was to learn huge amounts of content in short periods of time, on top of unrealistic expectations from clinical teachers.

*‘Trying not to look stupid because the standards are so high. And so you don’t want to say something that will make them think, “This guy doesn’t know what he’s talking about, he doesn’t really study, he’s just a waste of space”.’ (Arosh)*

Eight participants also articulated the emotional pressure on medical trainees, in that they witness and have to cope with confronting situations and negative outcomes.

*‘It has unique challenges, like raw human emotions, and just seeing trauma scenarios or even if you’re on the surgical ward and seeing drains or wounds, stuff like that can be a bit confronting.’ (Peter)*

Participants did not express these two pressures as problematic or demanding change.

Financial pressure was the third issue, discussed in depth by two participants, one of whom was from a rural location. Jarra and Abby said the expectation in medical school, due to the unpredictable timetable, is that you’re not employed, which they found unreasonable. They also believed the uncertainty medical students suffered with forcible relocation, sometimes annually, made university unnecessarily stressful and financially inaccessible, especially given the inadequate ‘rural scholarships’ allocated by their universities.

*‘I hate the uncertainty of it. I want to have at least one stable thing in my life, even being able to stay in the same area and knowing that I can keep the job that I have instead of having to look for a job.’ (Jarra)*

*‘You often have medical faculties telling students that they shouldn’t have a part-time job and that that’s incompatible with studying.’ (Abby)*

The fourth challenge for medical students was regarding gender. Gender seemed to affect the quality of teaching participants received, with female participants sometimes being made to feel uncomfortable, having fewer teaching opportunities, or being ignored, exclusively by male supervisors.

*‘He always referred to doctors as ‘him’ or ‘he’, so always in the male version… and often would do the same thing with nurses and other allied health, always referring to them as ‘she’. And so, immediately, myself and others in the class, felt like we didn’t belong in that classroom.’ (Alice)*

Gender discrimination ranged from a ‘quantity of low level things’ (Colette) to the most serious example of sexual harassment reported in this study, Abby’s friend having had a surgeon touch her thigh, ‘making it quite clear that to report it would be a blight on her future career’ (Abby).

Gender played a role in every female participant’s future decision-making. Female participants reported they would have to fight harder to succeed in certain specialties, and that certain training programs were inaccessible because of sexist culture or training requirements, such as having to repeat whole terms if maternity leave is taken. Six of the seven women used surgery as an example.

*‘Even the most benign aspects of sexism in the workplace, like being constantly reminded that I need to factor my future family into career choices, far beyond that of which my male peers receive or face, is pretty demotivating… When I think of things like specialties I’m leaning towards, I do actively wonder if things like not being interested in surgery isn’t a decision I came to by myself because of my interest, or because it has been consistently reinforced to me that that’s not an area where I would be welcomed or valued, and where I’d have to fight even harder to make it just because I’m a woman. I think those kinds of pressures weigh very heavily on the minds of women.’ (Abby)*

Two female participants from two different medical schools also noted gender played a role in the content of the curriculum, believing there to be excessive focus on normal male physiology and male study populations.

*‘All the women were a little bit frustrated with the time we spent on how normal male physiology works, but not a lot on how female physiology works… We didn’t have a “This is how normal menstruation works” lecture, whereas, as a girl, I know how that works. As a guy, you’re going to be in trouble.’ (Lauren)*

One queer-identifying participant noted similar lack of diversity, criticising the emphasis on heteronormative conceptions of health.

*‘Teaching is very hetero in its guidance. There’s very little discussion on how to deal with queer or gender queer people … I think it would be rare for us to be given a fake patient who is gay, facing a particular challenge relevant to health and being gay, or is incidentally gay presenting with something completely irrelevant.’ (Theo)*

To a lesser extent, racial discrimination was believed to be a stressor for some students, although not by the ethnic participants in this sample. The one international participant, Arosh, had not experienced any racism, while New Zealand-born Indian Sanjna, although she’d experienced racism, believed it was a societal problem no more prevalent in medicine than anywhere else. In fact, she reported ‘skinny, white, blonde’ female students were more likely to suffer sexual harassment because of conventional beauty standards. Five other participants said patients and older doctors commonly made racist comments, and that language barriers created a divide between international and local students and doctors.

*‘He would make, sort of, demographic jokes… and some of them weren’t appropriate.’ (Theo)*

### Mistreatment from patients

Eight participants reported witnessing or experiencing sexist or racist comments from patients, such as female medical students being assumed to be nurses, sexualising comments, or international students being criticised for their accents. However, two students said they did not know how to address these issues, which are usually brushed off, because not every moment can be a ‘disciplining, teaching moment’ (Abby) when you’re trying to manage the patient.

*‘Obviously I don’t enjoy patients being like, “I’ll have the pretty blonde one”, but can you really educate every single person who comes through the hospital?’ (Lily)*

### Quality of teaching

All ten participants provided some positive commentary on clinical teaching, with some participants emphasising that, for the most part, they had received good quality teaching.

However, several issues were raised regarding the quality of teaching. Firstly, six participants remarked on the indifference of teaching doctors, stating that many doctors appear to view teaching as a burden, reflected in their impatience and lack of effort. Being approachable and engaged in teaching was considered highly desirable, with ‘interest in students and other staff’ being the most commonly identified quality in participants’ role models, discussed by eight participants. Interestingly, Alice and Colette, both of whom had experienced rural teaching, believed lack of interest from teachers was less common in rural hospitals.

*‘I’ve had teams where it has been pretty clear that no one on the team [in a metropolitan hospital] was particularly interested in teaching or even having students… It made it very difficult for us to learn and for that to be a productive placement.’ (Abby)*

Abby and Sanjna accredited this to the lack of upskilling in senior doctors, who are expected to teach without any formal teaching training.

*‘Any level of responsibility at all and you’re expected to teach, and that’s the automatic default. There’s really no system finding people as being poor teachers or dangerous teachers.’ (Abby)*

This idea was often linked to the lack of standardisation in clinical teaching. Six participants, without prompt, expressed a lack of direction as one of the most stressful elements of learning, as there’s often a disconnect between examinable content and what senior doctors teach, and clarity is lacking regarding expected hours and level of knowledge.

*‘I think probably… a little bit more direction, in terms of clinical teaching. More clarity about expectations. Because it sometimes feels like you’re really unsure how many hours you’re supposed to be there, how much you’re supposed to know at different stages and what you should be doing with your time. And that’s been the number one stressful thing for me this year.’ (Colette)*

*‘There’s no standardisation in your clinical time. Registrars aren’t told what they’re meant to be doing with you. You’re often seen as a hindrance to the operation of the team. So you can either have really good clinical experiences, or you can be there doing nothing and it’s annoying and you’re expected to bear the burden of being the least efficient.’ (Sanjna)*

While participants did not report intellectual humiliation as a major problem in teaching, eight participants nevertheless described experiences of ‘grilling’, where senior doctors yelled at, cursed or belittled them for not performing tasks adequately or knowing answers to questions, usually in front of their peers. The participants stated these experiences made them uncomfortable and hindered their learning, often because they felt they could not approach these teachers with questions. They also believed this ‘baptism by fire’ (Abby) could damage individuals’ mental health.

*‘A particular lecturer … called a student out because he couldn’t answer a question, saying he was a ‘waste of space in the medical program’ because he could be taking up the place of a student who deserves and knows his stuff to be there. In front of the whole class.’ (Arosh)*

When asked what values are instilled in clinical teaching, participants identified academic values, such as dedication, communication and sacrifice. Three participants felt strongly that ethics, such as honesty and integrity, if taught at all, are taught superficially and in a tokenistic manner. One participant argued that any positive values emerging in medical students cannot be credited to any formal teaching, and the admission process is not rigorous enough to ensure all medical students have such values.

*‘I feel like sometimes there are values that they’re trying to enforce which are very important like honesty and integrity in medicine, but the way that they go about trying to teach you that is perhaps not the best… not really any formal or implied measuring of that or focus on that.’ (Lauren)*

*‘There’s certainly an increasing amount of lip service to things like diversity in leadership and being more aware of the medical workforce that we are and should be. But the extent to which that is really absorbed past medical school is, I think, hard to say… I think that the culture in medicine is stronger than the teaching in medicine and even the most aware and considerate medical student on graduating can be crushed into the general way of things pretty quickly.’ (Abby)*

In contrast, common positive characteristics of medical teaching included an emphasis on communication and patient-centred care, as well as tolerance and empathy for patients, respecting sociocultural factors contributing to their conditions.

*‘I think, probably, communication skills. That’s been a big thing. And not making assumptions about people.’ (Colette)*

## Discussion

This study provides insight into medical students’ experiences of mistreatment, and the impact it has on their wellbeing and clinical learning. Although participants reported only one serious instance of misconduct, they nevertheless identified some of the driving forces perpetuating mistreatment in medical school, as well as other challenges faced by medical students. The findings highlight problematic aspects of medical teaching and culture, and potential avenues for change and future research.

Participants often felt that intergenerational change was not occurring and that anti-bullying strategies have been ineffective because of the cyclical nature of mistreatment. This helps explain why mistreatment is entrenched in medical culture, and where intervention may be necessary. Wood [19] argued that the psychological qualities that allow some students to cope with abuse or ignore unwanted events perpetuate the problem. These students qualify, move into positions of authority within the medical workforce, and encourage the same behaviours and practice of non-reporting, not viewing these issues as problematic within their own training. For example, Australia’s Productivity Commission Report found 74% of medical students have been victims of and 86% have witnessed intellectual humiliation [20], in line with participants’ experiences and reinforcing their self-described need to be resilient. Scott & Caldwell [22] propose that intellectual humiliation forces students to align their values with those of their superiors, adjusting their professional ethical code in order to survive, and maintaining the dominant culture of medicine wherein victims become perpetrators. For this reason, more structural change may be needed, such as formal training of supervisors, to protect current medical students and break this cycle.

Several elements of medical culture were discussed by participants as problematic and potential contributors to mistreatment. Self-sacrifice and resilience were identified by participants as central to being a doctor. This plays into traditional medical paradigms; because of the vulnerability of patients and non-proprietary nature of the medical profession, doctors are bound by moral obligations to be altruistic, even when this compromises their own wellbeing [32]. Cohen [33], then President of the Association of American Medical Colleges, described ‘commitment to self-sacrifice’ as an essential attribute for acceptance into medical school. Major [34] attributed the emphasis on self-sacrifice to structural pressures in the healthcare system that require medical students to ‘act as professional and ethical chameleons’. Consequently, in an overburdened hospital, concealing minor errors and unconditionally agreeing with senior doctors are encouraged. Participants in this study, similarly, identified overworking and underreporting as products of self-sacrifice, propagating mistreatment.

Participants described the medical hierarchy as a pervasive feature of medical education and source of toxic workplace culture and underreporting. Similarly, a 2004 British qualitative study yielded the ‘hidden curriculum’ of medicine [35]. This was defined as an informal standard of learning that instills acceptance of hierarchy, compromise of ethical integrity and emotional neutralisation. These agendas may manifest as abuse and intellectual humiliation, leading to the perpetuation of the medical hierarchy at the cost of professionalism.

This study provides clear evidence to support the need for change in medical school and hospital environments. This may involve reforming the medical admission process, greater accessibility of reporting systems and emphasis on self-care, and formal workplace restructuring to prevent overworking, such as more interns allocated to a team.

Understanding how medical students approach reporting mistreatment was an important aspect of this study. Participants cited being labelled as ‘sensitive’ or a ‘troublemaker’ as barriers to reporting. The resilience participants identified as being necessary to cope with confronting emotional or physical situations may translate to professional reticence and underreporting, though this requires further research. Participants also lacked confidence in the anonymity of reporting systems and certainty of repercussions for troublesome personalities. These results reinforce Australian studies by the AMA [17] and Scott & Caldwell [22], who found less than one third of victims reported abuse in medical school. They cited fear of reprisal, lack of confidence in the reporting process, fear of impact on career, and cultural minimisation of the problem as reasons medical trainees do not report mistreatment. These findings align with a Canadian study that found, of students who had reported incidents, only 35.9% were satisfied with the outcome [36].

An array of solutions were suggested by participants to address unprofessional behaviour. For example, one participant mentioned the Vanderbilt Coworker Observation Reporting System (CORS) which aims to address disrespectful and unsafe physician behaviour with scaled consequences, escalating from an ‘informal’ cup of coffee intervention to ‘disciplinary’ intervention [37]. Piloting a similar framework in Australian medical schools and hospitals could potentially encourage utilisation of reporting systems, guarantee confidentiality, and assure outcomes, all of which were identified by participants as desirable to counter underreporting.

All seven female participants felt gender played a problematic role in their clinical teaching or future decision-making. They felt medicine was male-centric, in both the quality of teaching women received and curriculum itself. Female participants stated they had received fewer opportunities, were ignored by male supervisors, or felt uncomfortable because doctors were always referred to as ‘him’ or ‘he’ by physicians. Beyond Blue’s 2013 Australian survey, which included 1811 respondents, showed that 20% of medical students had contemplated suicide in the last 12 months, but 25% for female medical students [38]. However, this is the extent of academic literature regarding Australian female medical students’ experiences and perceptions of mistreatment. Because this was a key issue raised in this study, there is scope for further research into gendered experiences in clinical teaching.

Participants described a toxic workplace culture, including ‘servant’ behaviour and gendered mistreatment, which made participants and their peers feel uncomfortable, unsafe, and unhappy. The practice of ‘pimping’ students – an American term meaning excessive, inappropriate questioning of students and trainees that produces shame and humiliation– was also a stressor for participants [39]. Academic literature has well established that medical students who report mistreatment are more likely to experience lower career satisfaction including questioning their chosen profession, increased likelihood of drop out, substance abuse, anxiety, reduced self-esteem, higher levels of stress, depression and suicidality [15, 19, 36, 40, 41]. The literature also suggests that medical students’ experiences may differ by chosen specialty [15].

Six participants also reported that patient outcomes are compromised by overworking and mistreatment, factors like sleep and adequate family time being essential to better mental health and, consequently, quality of patient care. Disruptive workplace behaviour has been shown to have negative impacts on team collaboration and communication efficiency, diminishing staff performance and morale, and contributing to trainees feeling forced to cope with clinical issues beyond their training [17, 23, 42]. Similarly, a US staff survey showed that 71% of respondents (doctors and nurses) believed unprofessional behaviour contributed to medical errors, with 27% stating it had contributed to a patient’s premature death [43]. This is supported by a 2015 randomised blinded trial showing that medical teams treated ‘rudely by an expert observer’ performed significantly worse in a paediatric emergency simulation [44]. Furthermore, higher staff absenteeism due to stress and workplace dissatisfaction impacts continuity of patient care and increases the workload of other staff [43]. This study contributes to this body of literature necessitating structural change to address toxic medical culture in order to maximise quality of patient care and student wellbeing.

This study had some limitations. Firstly, the inclusion criteria purposefully sampled people who had experienced or witnessed mistreatment, which may have influenced the study’s results as participants were more likely to have strong views about mistreatment in clinical settings. Secondly, participants were only eligible if they attended a Sydney-based medical school. However, six of the participants had rural clinical experience, and discrepancies between rural and metropolitan teaching were often raised. There is scope for future research comparing rural and metropolitan teaching and working environments. Finally, as a small qualitative study the results may not be generalisable to a wider population.

Despite these limitations, this study provides a strong qualitative foundation for understanding the challenges faced by medical students, and how these obstacles, such as those preventing incident reporting, can be overcome. More clarity surrounding reporting systems, reinforcing their confidentiality and guaranteeing outcomes are some ways in which medical students may feel safer and more supported in their training. Additionally, providing participants with more structured clinical teaching, including enforcing formal learning objectives and supervisor training, may alleviate the anxiety and inappropriate teaching currently problematic in unorganised clinical teaching.

Furthermore, this study identifies concerns of medical students for which there may be scope for future research. These include inclusion of diversity in teaching, the medical admission process, and ethics and values in the medical curriculum.

## Conclusion

Given the persistent nature of these highly problematic behaviours in clinical workplaces [13, 16, 17], understanding how witnessing or experiencing clinical misconduct impacts medical students’ learning and wellbeing is important. Mistreatment of medical students has tangible consequences for future doctors, affecting their mental and physical wellbeing, as well as for wider society due to the implications on quality of patient care. This issue is therefore highly relevant. Structural change is needed to remedy the mistreatment institutionalised in medical culture and teaching.

## Data Availability

As these data are qualitative interviews, raw data will not be made available so as to protect participant confidentiality.

## Appendix A

**Table 2:** Definitions of Bullying, Harassment and Sexual Harassment [12]

Bullying	Bullying is unreasonable and inappropriate behaviour that creates a risk to health and safety. It is behaviour that is repeated over time or occurs as part of a pattern of behaviour. Such behaviour intimidates, offends, degrades, insults or humiliates. It can include psychological, social, and physical bullying.
Harassment	Harassment is unwanted, unwelcome or uninvited behaviour that makes a person feel humiliated, intimidated or offended, Harassment can include racial hatred and vilification, be related to a disability, or the victimisation of a person who has made a complaint.
Sexual Harassment	Sexual harassment is defined as unwelcome sexual advances, request for sexual favours and other unwelcome conduct of a sexual nature, by which a reasonable person would be offended, humiliated or intimidated. Sexual harassment may include, but is not limited to: leering; displays of sexually suggestive pictures, videos, audio tapes, emails & blogs, etc., books or objects; sexual innuendo; sexually explicit or offensive jokes; graphic verbal commentaries about an individual’s body; sexually degrading words used to describe an individual; pressure for sexual activity; persistent requests for dates; intrusive remarks, questions or insinuations about a person’s sexual or private life; unwelcome sexual flirtations, advances or propositions; and unwelcome touching of an individual, molestation or physical violence such as rape.

## Appendix B

### Interview domains

#### Introduction

*Participant selects pseudonym*

*Basic demographics collected – age, education level, ethnicity*

#### Clinical experiences

- Have you had any experiences of bullying, harassment or humiliation in your clinical teaching?
  - ○ What form did this experience take? i.e. verbal, sexual, physical
  - ○ In what settings did these occur?
  - ○ Who was the perpetrator? i.e. JMO, consultant, surgeon, fellow medical student, nurse, allied health practitioner, patient
- Do you feel that medicine is a difficult profession? And if so, for what reasons? [Prompt – emotionally draining? intellectually challenging? pressures from superiors? over-worked?]
- Do you feel that, because of your gender, your experiences of clinical teaching have been different? [Prompts – have you ever felt stereotyped because of your gender e.g. as to what specialties appeal/are more appropriate, confused for a nurse/junior by patients; do you feel limited in medicine because of your gender?]
- Do you feel that, because of your ethnicity, your experiences of clinical teaching are different?
- Generally, how do you feel about the structure of medicine? [Prompts – do you feel there is a strong hierarchy in medicine? do you find senior doctors approachable? do you think the hierarchy is essential to teaching/practice?]
- Who are your role models in medicine and why? [Prompt – who were some of the best clinical teachers and why?]

#### Responses to clinical experiences

- Do you think that these clinical experiences are beneficial to your learning/practice as future clinician?
- What values do you feel are instilled in medical students in clinical teaching? [Prompts – compassion? work satisfaction? emotional neutralisation? acceptance of hierarchy?]
- Do you feel you have avenues of recourse if you take issue with clinical teaching? [if so, have you ever used them? What were the outcomes?]
- Have you discussed these experiences with other medical students? If so, were these experiences universal? [Prompt – which particular demographics share these experiences most? What is the reputation of clinical teaching among medical students?]

#### Issues & potential avenues for change

- Do you feel there are issues with clinical teaching/structure of medicine?
- Have you suffered any consequences of these clinical experiences? [Prompt – mental health? stress? influenced your decisions about clinical schools, specialties?]
- Do you think the medical hierarchy is a necessary part of the profession?
- What changes would you like to see in medicine/clinical teaching? Why do you think these changes are necessary?
  - What are some ways you believe such change could be achieved?
- Have you ever been involved in advocacy for such change? If not, why?

#### Final

- Is there anything more you’d like to tell me before we finish, anything that you consider of particular importance that we haven’t covered or that you’d like to explain more?

## Abbreviations

AMA: Australian Medical Association
JAMA: Journal of the American Medical Association
US: United States
